# Lactation Associated Genes Revealed in Holstein Dairy Cows by Weighted Gene Co-Expression Network Analysis (WGCNA)

**DOI:** 10.3390/ani11020314

**Published:** 2021-01-27

**Authors:** Yongliang Fan, Abdelaziz Adam Idriss Arbab, Huimin Zhang, Yi Yang, Mudasir Nazar, Ziyin Han, Zhangping Yang

**Affiliations:** 1 College of Animal Science and Technology, Yangzhou University, Yangzhou 225009, China; dx120170088@yzu.edu.cn (Y.F.); arbabtor@yahoo.com (A.A.I.A.); minmin-911@163.com (H.Z.); drmudasirnazar457@gmail.com (M.N.); ZiyinHan@126.com (Z.H.); 2 Joint International Research Laboratory of Agriculture & Agri-Product Safety, Ministry of Education, Yangzhou University, Yangzhou 225009, China; 3 Jiangsu Co-innovation Center for the Prevention and Control of Important Animal Infectious Diseases and Zoonoses, Yangzhou University College of Veterinary Medicine, Yangzhou 225009, China; yangyi@yzu.edu.cn

**Keywords:** WGCNA, dairy cows, mammary gland, lactation stage, milk composition, milk yield

## Abstract

**Simple Summary:**

Weighted gene coexpression network analysis (WGCNA) is a novel approach that can quickly analyze the relationships between genes and traits. In the past few years, studies on the gene expression changes of dairy cow mammary glands were only based on transcriptome comparisons between two lactation stages. Few studies focused on the relationships between gene expression of the dairy mammary gland and lactation stage or milk composition in a lactation cycle. In this study, we detected milk yield and composition in a lactation cycle. For the first time, we constructed a gene coexpression network using WGCNA on the basis of 18 gene expression profiles during six stages of a lactation cycle by transcriptome sequencing, generating 10 specific modules. Genes in each module were performed with gene ontology (GO) annotation and Kyoto Encyclopedia of Genes and Genomes (KEGG) pathway analysis. Module–trait relationship analysis showed a series of potential candidates related to milk yield and composition. The current study provides an important theoretical basis for the further molecular breeding of dairy cows.

**Abstract:**

Weighted gene coexpression network analysis (WGCNA) is a novel approach that can quickly analyze the relationships between genes and traits. In this study, the milk yield, lactose, fat, and protein of Holstein dairy cows were detected in a lactation cycle. Meanwhile, a total of 18 gene expression profiles were detected using mammary glands from six lactation stages (day 7 to calving, −7 d; day 30 post-calving, 30 d; day 90 post-calving, 90 d; day 180 post-calving, 180 d; day 270 post-calving, 270 d; day 315 post-calving, 315 d). On the basis of the 18 profiles, WGCNA identified for the first time 10 significant modules that may be related to lactation stage, milk yield, and the main milk composition content. Genes in the 10 significant modules were examined with gene ontology (GO) annotation and Kyoto Encyclopedia of Genes and Genomes (KEGG) pathway analysis. The results revealed that the galactose metabolism pathway was a potential candidate for milk yield and milk lactose synthesis. In −7 d, ion transportation was more frequent and cell proliferation related terms became active. In late lactation, the suppressor of cytokine signaling 3 (*SOCS3*) might play a role in apoptosis. The sphingolipid signaling pathway was a potential candidate for milk fat synthesis. Dairy cows at 315 d were in a period of cell proliferation. Another notable phenomenon was that nonlactating dairy cows had a more regular circadian rhythm after a cycle of lactation. The results provide an important theoretical basis for the further molecular breeding of dairy cows.

## 1. Introduction

Bovine milk is not only the sole nutrition source of newborn dairy cows, but also an important source of proteins, sugars, lipids, and other nutrients for humans [1,2]. The synthesis, secretion, and storage of milk can only be carried out in the mammary glands; hence, milk yield and quality depend on the mammary glands [3]. A lactation cycle is divided into different lactation stages according to the daily milk yield of dairy cows [4,5,6]. Recently, a few studies focused on mammary gland gene expression at limited lactation stages to clarify the variation of gene expression profiles between different lactation stages [7,8,9]. 

The exploration of gene expression in dairy cow mammary glands at different lactation periods is accompanied by upgrading the technology and analysis method. The measurement of gene expression underwent changes from quantitative PCR to DNA microarray and then to transcriptome sequencing [10,11,12]. Gradually, gene ontology (GO) annotation and pathway analysis replaced the interaction analysis of limited differentially expressed genes (DEGs) [10,11,12,13]. Studies using transcriptome sequencing as a means mainly focused on comparing the gene expression of dairy cow mammary glands between two lactation stages [8,9]. Almost none comprehensively analyzed alternations in the gene expression of mammary glands in a lactation cycle combined with metabolomics and transcriptome sequencing.

With the advent of high-throughput technology, methods such as a single gene differential expression analysis or cluster analysis often miss a lot of useful information when faced with a complex network composed of a large amount of DNA, RNA or protein. Therefore, a biological network analysis method emerged on the basis of global gene expression patterns [14,15,16,17]. One of the most useful methods is weighted gene coexpression network analysis (WGCNA), which can be used in unweighted correlation networks to alleviate multiple detection problems from big data analysis. WGCNA is used in some studies on diseases and plants [14,18,19,20]. It has not been reported on the application of WGCNA in the gene expression of dairy cow mammary gland up to now.

This study constructed for the first time a coexpression network of mammary gland genes at six different stages of the lactation cycle on transcriptome sequencing data and WGCNA, which then divided modules and mined specific modules. When combined with the data about milk yield and composition, key terms and pathways in specific modules were analyzed to reveal the underlying molecular mechanism in the relationship of transcriptome and metabolite profiling.

## 2. Materials and Methods

### 2.1. Ethics Statement

This study was approved by the Institutional Animal Care and Use Committee (IACUC) of the Yangzhou University Animal Experiments Ethics Committee (Permit Number: SYXK (Su) IACUC 2016–0019). All animals were fed according to national regulations.

### 2.2. Sample Collection

A total of 33 healthy Holstein dairy cows in their second lactation period were selected from the Yangzhou University experimental farm. The 33 animals with similar body weights were fed with total mixed ration (TMR) [9]. Milk samples of the 33 dairy cows were collected from the left anterior mammary region on early lactation (30 days after calving (30 d)), peak lactation (90 days after calving (90 d)), mid lactation (180 days after calving (180 d)), and late lactation (270 days after calving (270 d)), and transported on ice to the lab within 2 h for the determination of milk composition. Three Holstein dairy cow (A, B, C) mammary glands were collected using surgical methods at six lactation stages (−7 d, 30 d, 90 d, 180 d, 270 d, 315 d) [21]. In detail, the biopsy site was shaved and disinfected with 75% ethanol, and 1 mL procaine was injected subcutaneously. Then, the disinfected biopsy site was incised along the midpoint to collect the parenchymal mammary glands. Lastly, the parenchymal mammary glands were transferred to sterile tubes and frozen in liquid nitrogen until RNA isolation.

### 2.3. Milk Composition Detection

Milk fat, protein and lactose concentrations were determined using mid-infrared spectrometry (MilkoScan Minor, Foss Analytics, Hillerød, Denmark) [22]. The daily milk yield of individual dairy cows was recorded in the experimental period.

### 2.4. RNA Extraction and cDNA Library Analysis

The mirVana™ miRNA Isolation Kit (Ambion-1561) was used for the extraction of total RNA in each sample. Then, RNA integrity was measured on the Agilent 2100 Bioanalyzer (Agilent Technologies, Santa Clara, CA, USA) [14,23]. The samples with RNA Integrity Number (RIN) of greater than or equal to 7 were analyzed subsequently.

The TruSeq Stranded mRNA LT Sample Prep Kit (Illumina, RS-122-2101) was used for the construction of 18 cDNA libraries [24], and the Illumina sequencing platform (HiSeqTM 2500) was used to sequence these libraries, generating 125 bp paired-end reads [25,26]. Raw data underwent recognition analysis to obtain original sequencing. To generate the clean reads, raw reads were purified by removing ploy-N contained and low-quality reads. Clean reads were mapped to reference bovine genome UMD3.1 [27]. 

Datasets were obtained from the NCBI Sequence Read Archive (SRA) (https://www.ncbi.nlm.nih.gov/sra) with accession number SRR12136243 (−7 d), SRR12149783 (30 d), and SRR12149843 (90 d), and the Genome Sequence Archive in the BIG Data Center, Beijing Institute of Genomics (BIG), and the Chinese Academy of Sciences under accession number CRA002742 (180 d, 270 d and 315 d), and are publicly accessible at http://bigd.big.ac.cn/gsa.

### 2.5. Weighted Gene Coexpression Network Analysis

#### 2.5.1. Gene Coexpression Network Construction

Gene expression was calculated using the fragments per kilobase of exon model per million mapped fragments (FPKM) method [28]. HtSeq-count 0.9.1 was used for the generation of the read counts of each gene. The expression patterns of genes were examined with principal component analysis (PCA). To demonstrate the dynamics of genes identified in Holstein dairy cow mammary glands at different lactation stages, coexpression modules were constructed using the WGCNA package tool [29]. First, genes with a 0 FPKM in all samples were removed from analysis [30,31]. Pearson’s correlation coefficient matrix was established the between gene pairs of all samples. Next, the influence of noise and spurious associations was minimized by transforming the adjacency matrix into a topological overlap matrix (TOM). Then, the generation of the hierarchical clustering of disparate matrices relied on the hierarchical clusters (HCLUST) function. Lastly, these cluster trees were divided using Dynamic Tree Cut with relevant parameter minModuleSize = 36. In this process, genes with similar expression patterns could be combined in the same branch, and each branch represented a coexpression module.

#### 2.5.2. Interaction Analysis of Coexpression Modules

The exploration of the interactions between coexpression modules utilized a heat map plot showing TOM according to the expression levels of genes [32]. In the heat map plot, color depth was related to overlapping degree. Light color indicated low overlap and high independence, and dark red indicated high overlap and low independence. In addition, eigengene connectivity was analyzed to discover the interactions of the coexpression modules [33]. Modules with only high independence and difference in the connectivity effect compared to other modules could be used for subsequent module–trait relationship analysis.

#### 2.5.3. Module–Trait Relationships Analysis

Module eigengenes calculated correlation coefficients between modules and external traits to identify modules with biological significance [34]. Module–trait relationships were visualized in heat maps. The intramodular connectivity of genes was calculated, and genes with higher connectivity tended to be hub genes [14]. Genes in critical modules were mapped to protein–protein interaction (PPI) networks of cattle to reveal the biological activities and interactive relationships of proteins encoded by these genes. PPI networks were visualized with Cytoscape software (3.7.1). In these PPI networks, yellow nodes expressed the hub genes in each module.

Kyoto Encyclopedia of Genes and Genomes (KEGG) pathway analysis and GO annotation were performed to investigate the biological functions of genes in critical modules. Significant KEGG pathway profiles and GO terms were visualized by Cytoscape software (3.7.1) on the basis of network analysis of ClueGO/CluePedia inferred from molecular complex detection (MCODE) clusters [35,36,37]. Pathways and terms with a *p*-value of less than 0.05 were identified as significant pathways.

### 2.6. Sequencing Data Validation by qRT-PCR

Ten genes were selected to validate transcriptome sequencing data. The Light Cycler^®^ 480 System (Roche, USA), TB Green^®^ Premix Ex Taq™ II Kit (TaKaRa, RR820), and specific primers (Table S1) were used for amplifications. Amplification was performed with three biological repetitions and each biological repetition carried out three technical replicates. Relative expression was calculated by the 2^−ΔΔCt^ method using *β-actin* as the internal reference.

### 2.7. Statistical Analysis

Statistical analyses of milk yield and composition were performed by two-way analysis of variance (ANOVA) using the Tukey–Kramer adjusted generalized linear model (GLM) procedures of Statistical Analysis Software (SAS) 9.4 (SAS Institute, Cary, NC, USA), and presented as mean ± standard error (SE). qRT-PCR results are shown using Prism 6.02 (GraphPad, San Diego, CA, USA) and presented as mean ± standard deviation (SD). Statistical significance (*p* < 0.05) was determined using Student’s t-test.

## 3. Results

### 3.1. Milk Yield and Component Results

Milk yield and component data are shown in Table 1. Milk yield and lactose content were significantly increased from 30 to 90 d, and then constantly decreased from 90 to 270 d (*p* < 0.05). Milk fat content continuously increased during the lactation period. Milk protein content was stable from 30 to 90 d (*p* > 0.05) and then consistently rose with the increase in lactation days (*p* < 0.05).

### 3.2. Transcriptome Sequencing Data Analysis

To comprehensively reveal the dynamic changes of gene related lactation, a total of 18 cDNA libraries were generated from three Holstein dairy cow (A, B, C) mammary glands at six lactation stages. More than 70.01 GB raw bases and 0.56 GB raw reads were generated in each library (Table S2). After quality control, the effectiveness rates of bases and reads were 97.27% and 97.31%, respectively. Q30 ratio was higher than 95.84% and GC content was above 47.50%. Unique match reads were higher than 90.64% when clean reads were aligned to the bovine reference genome (Table S3).

### 3.3. Gene Coexpression Network Construction

**Figure 1:**
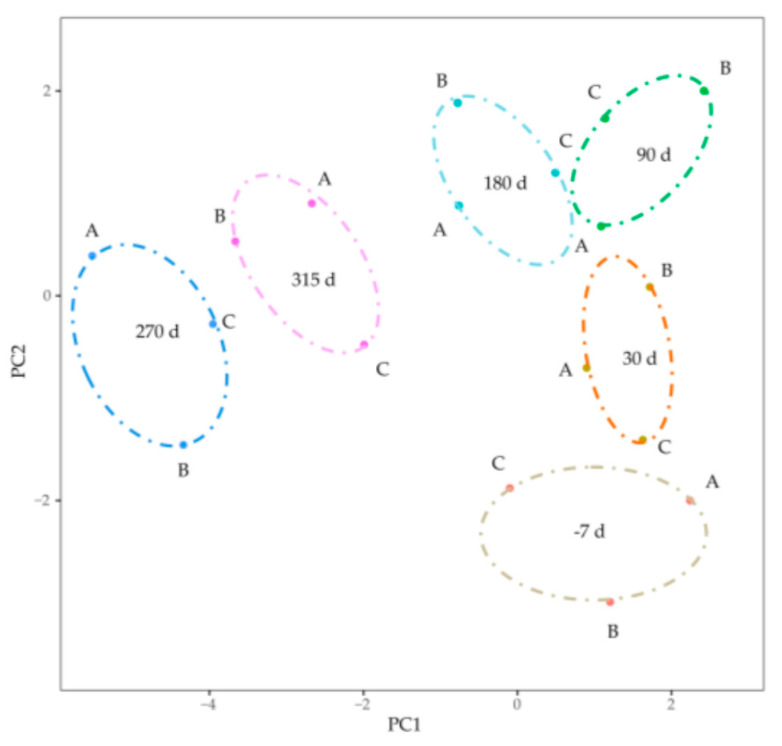
Visualizing the expression distribution of global genes by principal component analysis. The dots marked with A, B and C represented mRNAs form the three Holstein dairy cows. The ovals with different color represented different lactation stages.

**Figure 2:**
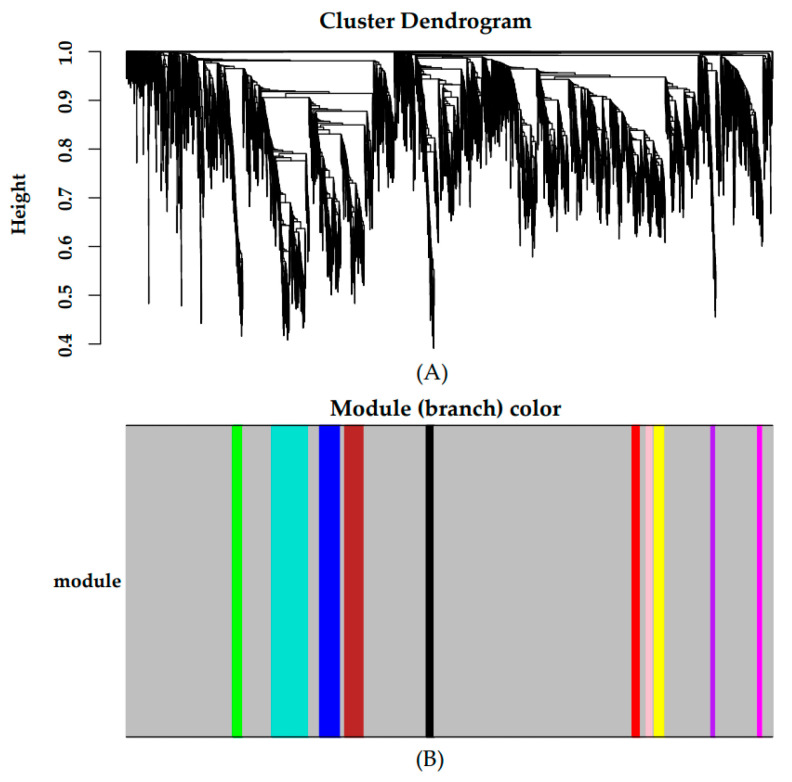
Gene cluster dendrograms and module detecting. (**A**) Clustering of genes based on the topological overlap. (**B**) The merged modules with similar expression patterns.

**Figure 3:**
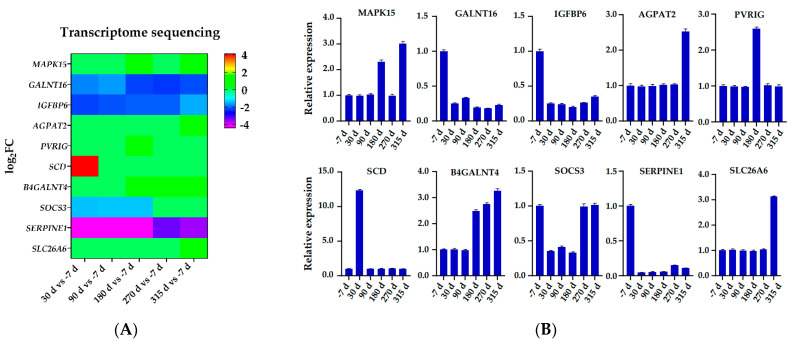
Verification transcriptome sequencing data using qRT-PCR. (**A**) Transcriptome sequencing data based log2-fold change expression. (**B**) qRT-PCR-based relative expression profiles.

Gene abundances were estimated on basis of FPKM. PCA was used to visualize the expression distribution of global genes and the result showed that genes in a cluster were from three dairy cows at the same lactation stage, which indicated that differences in gene expressions appeared at different lactation stages (Figure 1). Coexpression modules were constructed using the WGCNA package tool to explore the expression patterns of genes detected in 18 mammary gland samples from six lactation stages. First, we confirmed that the correlation coefficient between gene pairs was 0.80. Then, to make the network meet scale-free distribution, an appropriate weight of 18 and independence of 0.85 were selected using the soft threshold tool of the WGCNA package (Figure S1). A total of 11 modules composed of genes with similar expression patterns were generated by cutting a dynamic hierarchical tree using the HCLUST function (Figure 2). The size of 11 coexpression modules marked with different colors depended on the number of genes that they contained (Table 2). In addition, 10 genes were selected for qRT-PCR to validate the reliability of transcriptome sequencing data (Figure 3). The expression patterns of the 10 genes in the qRT-PCR results were highly consistent with transcriptome sequencing data.

### 3.4. Interaction Analysis of Coexpression Modules

**Figure 4:**
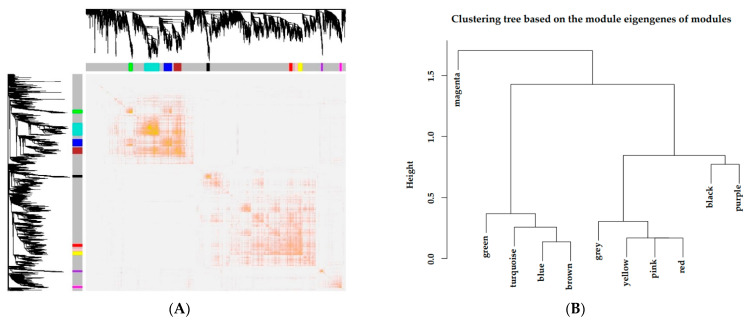
Independence inspection of gene coexpression modules. (**A**) Topological overlapping heat map. Both rows and columns in the figure represented the single gene. Light color indicated low overlap, and dark red indicated high overlap. (**B**) Correlation clustering of expression matrix gene coexpression modules.

According to the expression levels of genes, a heat map plot (Figure 4A) showing TOM was generated to explore interactions between the eleven coexpression modules. There were no differences between modules except for some high-brightness areas. In addition, eigengene connectivity was analyzed to discover the interactions of the coexpression modules. Eigengene cluster analysis showed that the eleven clusters divided into two clusters (Figure 4B), in which a cluster contained a magenta module and the other cluster contained the 10 remaining modules (red, purple, black, yellow, green, pink, turquoise, blue, brown and grey modules).

### 3.5. Module–Trait Relationships Analysis

**Figure 5:**
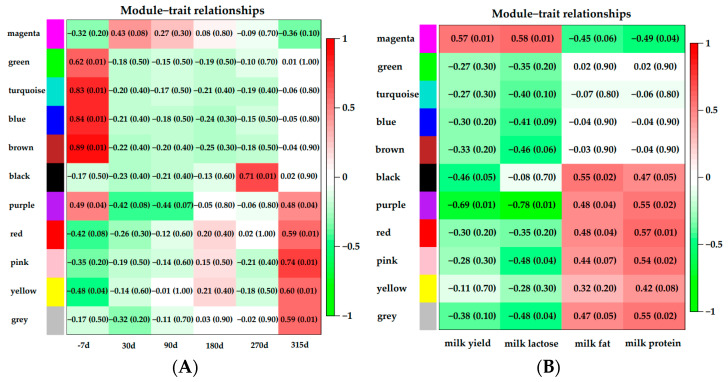
Module–trait relationship analysis. Each row corresponded to a module characteristic gene (eigengene), and each column corresponded to a trait. (**A**): the correlations of modules and lactation stages; (**B**): the correlations of modules and milk yield or component. Each cell contained a corresponding correlation and *p*-value.

The correlations of modules and lactation stages are shown in Figure 5A. There was a significant positive correlation between −7 d and five modules: green (*p* = 0.01, r = 0.62), turquoise (*p* = 0.01, r = 0.83), blue (*p* = 0.01, r = 0.84), brown (*p* = 0.01, r = 0.89) and purple (*p* = 0.04, r = 0.49) modules. Late lactation had a significant positive correlation with the black module (*p* = 0.01, r = 0.71). Modules that were highly correlated with the dry period were the purple (*p* = 0.04, r = 0.48), red (*p* = 0.01, r = 0.59), pink (*p* = 0.01, r = 0.74) and yellow (*p* = 0.01, r = 0.60) modules.

Correlations of modules and milk yield or component are shown in Figure 5B. One module (magenta, *p* = 0.01, r = 0.57) was correlated with milk yield. Milk lactose content showed significant positive correlation with the magenta module (*p* = 0.01, r = 0.57). Three modules were significantly associated with milk fat: the black (*p* = 0.02, r = 0.55), purple (*p* = 0.04, r = 0.48), and red (*p* = 0.04, r = 0.48) modules. The modules that were correlated with milk protein content were the black (*p* = 0.05, r = 0.47), purple (*p* = 0.02, r = 0.55), red (*p* = 0.01, r = 0.57) and pink (*p* = 0.02, r = 0.54) modules.

### 3.6. Functional Enrichment Analysis of Critical Modules

**Figure 6:**
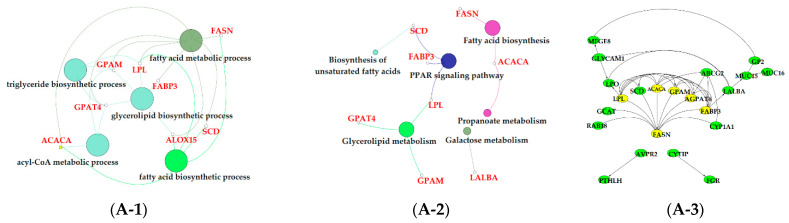

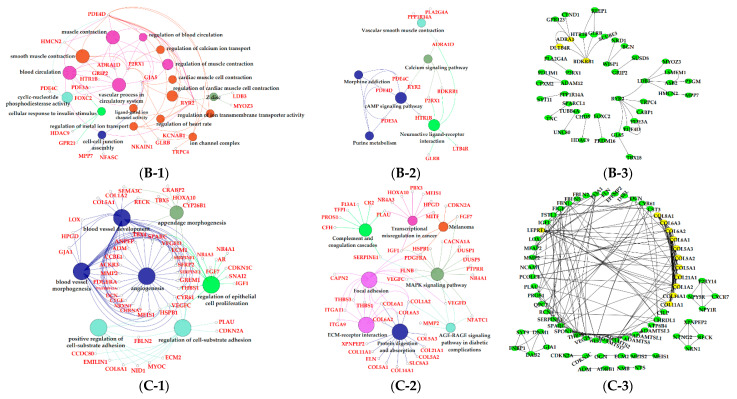
Functional analysis of genes in magenta, green and turquoise modules. Gene ontology (GO) analysis of genes in magenta (**A-1**), green (**B-1**) and turquoise (**C-1**) modules. Kyoto Encyclopedia of Genes and Genomes (KEGG) pathway analysis of genes in magenta (**A-2**), green (**B-2**) and turquoise (**C-2**) modules. The significant GO terms and KEGG pathways were visualized using Cytoscape software based on network analysis of ClueGO/CluePedia inferred from MCODE clusters. The size of nodes indicated the *p*-value. The color of nodes represented the functional group that they belonged to. The names of important pathways were in black bold characters. The protein–protein interaction (PPI) networks showed the interactions of genes in the magenta (**A-3**), green (**B-3**) and turquoise (**C-3**) modules, respectively. Each node represented a gene and the yellow nodes expressed the key genes in each module.

**Figure 7:**
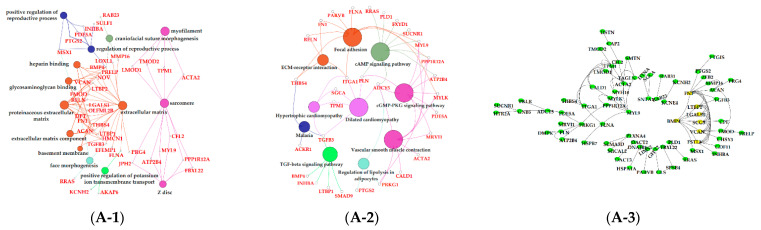

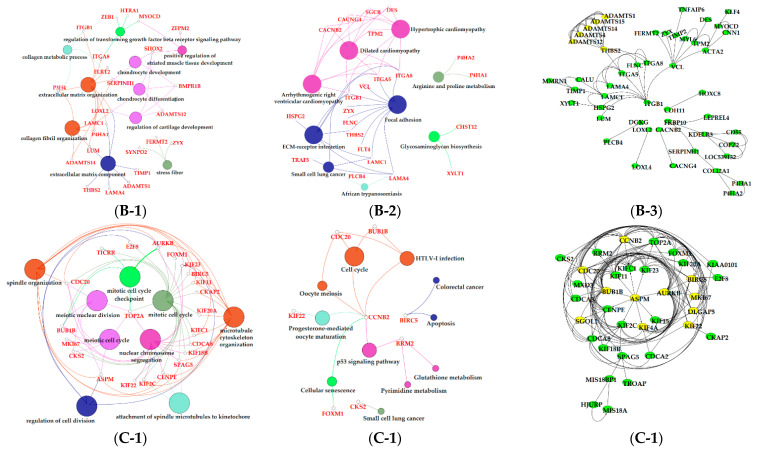
Functional analysis of genes in bule, brown and purple modules. Gene ontology (GO) analysis of genes in bule (**A-1**), brown (**B-2**) and purple (**C-1**) modules. Kyoto Encyclopedia of Genes and Genomes (KEGG) pathway analysis of genes in bule (**A-2**), brown (**B-2**) and purple (**C-2**) modules. The significant GO terms and KEGG pathways were visualized using Cytoscape software based on network analysis of ClueGO/CluePedia inferred from MCODE clusters. The size of nodes indicated the *p*-value. The color of nodes represented the functional group that they belonged to. The names of important pathways were in black bold characters. The protein–protein interaction (PPI) networks showed the interactions of genes in the bule (**A-3**), brown (**B-3**) and purple (**C-3**) modules, respectively. Each node represented a gene and the yellow nodes expressed the key genes in each module.

**Figure 8:**
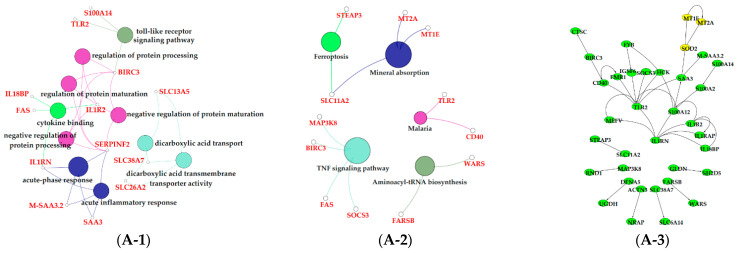

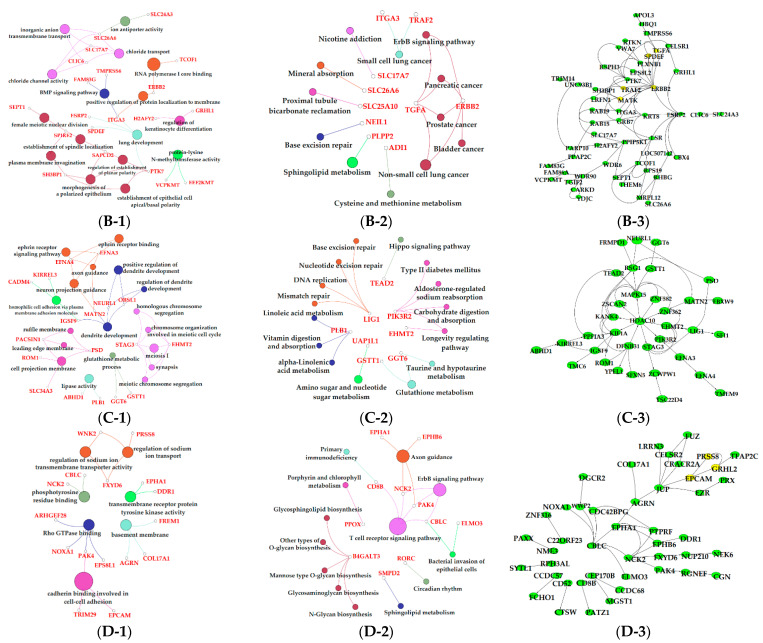
Functional analysis of genes in black, red, pink and yellow modules. Gene ontology (GO) analysis of genes in black (**A-1**), red (**B-1**), pink (**C-1**) and yellow (**D-1**) modules. Kyoto Encyclopedia of Genes and Genomes (KEGG) pathway analysis of genes in black (**A-2**), red (**B-2**), pink (**C-2**) and yellow (**D-2**) modules. The significant GO terms and KEGG pathways were visualized using Cytoscape software based on network analysis of ClueGO/CluePedia inferred from MCODE clusters. The size of nodes indicated the *p*-value. The color of nodes represented the functional group that they belonged to. The names of important pathways were in black bold characters. The protein–protein interaction (PPI) networks showed the interactions of genes in black (**A-3**), red (**B-3**), pink (**C-3**) and yellow (**D-3**) modules, respectively. Each node represented a gene and the yellow nodes expressed the key genes in each module.

The results of GO enrichment and KEGG pathway analysis are shown in Figure 6, Figure 7 and Figure 8. The magenta module showed significantly high correlation with lactose synthesis and milk yield. In the magenta module, genes were significantly enriched in the galactose metabolism pathway (KEGG: 00052; Figure 6A-2, File S2). The green module contained five terms involved in ion transportation, namely, the regulation of the metal ion transport term (GO: 0010959), the ligand-gated ion channel activity term (GO: 0015276), the ion channel complex term (GO: 0034702), the regulation of ion transmembrane transporter activity term (GO: 0032412), and the regulation of calcium ion transport term (GO: 0051924; Figure 6B-1, File S1). In the turquoise module, genes were significantly enriched in the regulation of the epithelial cell proliferation term (GO: 0050678; Figure 6C-1, File S1). The blue module contained the positive regulation of potassium ion transmembrane transport term (GO: 1901381; Figure 7A-1, File S1). In the brown module, genes were significantly enriched in the extracellular matrix component term (GO: 0044420; Figure 7B-1, File S1). In the purple module, mostly significant terms were associated with cell proliferation, such as the microtubule cytoskeleton organization term (GO: 0000226), the mitotic cell cycle term (GO: 0000278), the spindle organization term (GO: 0007051), the mitotic cell cycle checkpoint term (GO: 0007093), the attachment of spindle microtubules to kinetochore term (GO: 0008608), and the regulation of cell division term (GO: 0051302) (Figure 7C-1, File S1). The cell cycle pathway (KEGG: 04110) was also significantly enriched in the purple module (Figure 7C-2, File S2). In the black module, GO analysis showed that genes in the following terms were significantly enriched: the dicarboxylic acid transmembrane transporter activity term (GO: 0005310), the dicarboxylic acid transport term (GO:0006835), the regulation of protein processing term (GO: 0070613), the regulation of protein maturation term (GO: 1903317), the negative regulation of protein processing term (GO: 0010955), and the negative regulation of protein maturation term (GO: 1903318; Figure 8A-1, File S1). In the red module, significantly enriched pathways included the base excision repair pathway (KEGG: 03410), the sphingolipid signaling pathway (KEGG: 00600), and the glycosphingolipid biosynthesis pathway (KEGG: 00601; Figure 8B-2, File S2). In the pink module, genes were significantly enriched in the DNA replication pathway (KEGG: 03030), the base excision repair pathway (KEGG: 03410), the nucleotide excision repair pathway (KEGG: 03420), and the mismatch repair pathway (KEGG: 03430) (Figure 8C-2, File S2). In the yellow module, genes were significantly enriched in the circadian rhythm pathway (KEGG: 04710; Figure 8D-2, File S2).

## 4. Discussion

Traditional biological research on factors affecting life activities is usually based on exploring the molecular mechanisms of DNA, mRNA, or proteins [15]. The limitations of traditional biological research were exposed with the rapid development of sequencing technology, namely, it only has importance in revealing the genetic mechanism of specific traits, but it cannot fully and effectively mine biological significance contained in massive data [38,39,40,41]. WGCNA is an effective data mining method compared with other regulatory networks, modularizing large data sets to obtain coexpression modules with high biological significance on the basis of the similar expression patterns of genes [42]. In recent years, WGCNA has been applied to explore the life activity characteristics of humans and plants [14,18,19,20]. However, studies on cattle using the WGCNA method have never been reported. In the current study, we constructed a coexpression network of genes in Holstein dairy cow mammary glands at six different stages from a lactation cycle for the first time and found 10 specific modules associated with lactation stages, milk yield, lactose, fat, and protein content.

The magenta module showed positive correlations with milk yield and lactose content, respectively (Figure 5B). In addition, the milk lactose content and milk yield were positively correlated according to our findings and also those of previous studies [43,44]. Lactose is a major milk osmolyte which allows fluid to be drawn into milk secretory vesicles, influencing milk volume, and subsequently, requires a substantial amount of lactose [44,45]. Milk lactose synthesis uses glucose absorbed by bovine mammary epithelial cells (BMECs) as a substrate. Function analysis showed that the galactose metabolism pathway (KEGG: 00052) was significantly enriched in the magenta module (Figure 6A-2, File S2). In the galactose metabolism pathway, lactalbumin alpha (*LALBA*) as a mammary epithelial-specific protein is a subunit of lactose synthase, and regulates the synthesis and secretion of lactose [46]. Previous studies indicated that the abundance of *LALBA* in the mammary glands of lactating dairy cows was higher than that in the mammary glands of nonlactating dairy cows [8,47].

The green, turquoise, blue, brown, and purple modules were respectively positively correlated with −7 d. Ion transportation was more frequent in this period (Figure 5A). The positive regulation of the potassium ion transmembrane transport term (GO: 1901381) was activated in the blue module (Figure 7A-1, File S1). Five terms in the green module were involved in ion transportation, namely, the regulation of metal ion transport term (GO: 0010959), the ligand-gated ion channel activity term (GO: 0015276), the ion channel complex term (GO: 0034702), the regulation of ion transmembrane transporter activity term (GO: 0032412), and the regulation of calcium ion transport term (GO: 0051924; Figure 6B-1, File S1). Another characteristic of this period was that the proliferation of BMECs significantly increased [48]. In the purple module, mostly significant terms were associated with cell proliferation, such as the microtubule cytoskeleton organization term (GO: 0000226), the mitotic cell cycle term (GO: 0000278), the spindle organization term (GO: 0007051), the mitotic cell cycle checkpoint term (GO: 0007093), the attachment of spindle microtubules to kinetochore term (GO: 0008608), and the regulation of cell division term (GO: 0051302; Figure 7C-1, File S1). The cell cycle pathway (KEGG: 04110; Figure 7C-2, File S2) in the purple module and the regulation of epithelial cell proliferation term (GO: 0050678; Figure 6C-1, File S1) in the turquoise also contributed to BMECs proliferation during this period. The mammary gland is a dynamic organ that undergoes cycles of cell proliferation, differentiation, and apoptosis during adult life. The number of mammary gland cells is an important factor determining milk production and the newly formed cells are about 50% of the original cells in the mammary gland of dairy cows [49].

The black module was positively correlated with late lactation (270 d) (Figure 5A). The negative regulation of the protein processing term (GO: 0010955) and the negative regulation the of protein maturation term (GO: 1903318) were significantly enriched in this module (Figure 8A-1, File S1). This indicated that the mammary glands of dairy cows attenuated protein synthesis. The apoptosis and decrease in mammary gland cells are characteristics of mammary gland regression in late lactation [50], which is the reason why daily milk yield decreased in late lactation. A study confirmed that the suppressor of cytokine signaling 3 (*SOCS3*)-deficient mammary glands displayed an increase in epithelial apoptosis and tissue remodeling, indicating that *SOCS3* was a vital attenuator of pro-apoptotic pathways in developing mammary glands [51]. This was consistent with our findings that *SOCS3* regulated apoptosis during involution, which exhibited higher expression in late lactation and dry period versus early, peak, and mid lactation.

The purple, red, pink, and yellow modules were positively correlated with the dry period (315 d) (Figure 5A). Pathway analysis showed that genes in the purple module were significantly enriched in the apoptosis (KEGG: 04215) and cellular senescence (KEGG: 04218; Figure 7C-2, File S2); the apoptosis and decrease in mammary gland cells were continued in 315 d. Moreover, in the purple module, we found some terms and pathways associated with cell proliferation: the microtubule cytoskeleton organization term (GO: 0000226), the mitotic cell cycle term (GO: 0000278), the spindle organization term (GO: 0007051), the mitotic cell cycle checkpoint term (GO: 0007093), the attachment of spindle microtubules to kinetochore term (GO: 0008608), the regulation of cell division term (GO: 0051302; Figure 7C-1, File S1), and the cell cycle pathway (KEGG: 04110; Figure 7C-2, File S2) in the purple module. We speculated that the reason for cell proliferation during this period was that mammary glands were repairing themselves after a cycle of lactation [9]. Correspondingly, in the pink and red modules, pathways related to DNA replication were activated in the dry period, which contained the DNA replication (KEGG: 03030), base excision repair (KEGG: 03410), nucleotide excision repair (KEGG: 03420), and mismatch repair pathways (KEGG: 03430; Figure 8C-2, File S2). In the yellow module, genes were significantly enriched in the circadian rhythm pathway (KEGG: 04710; Figure 8D-2, File S2), meaning that the mammary glands of dairy cows had increased circadian rhythm in the dry period compared to that in the other periods.

The black and red modules were positively correlated with dairy fat (Figure 5B). In the red module, the sphingolipid signaling (KEGG: 00600) and glycosphingolipid biosynthesis (KEGG: 00601) were also found to be potential candidates for lipid metabolism in lactation mammary glands (Figure 8B-2, File S2). Sphingolipids are a major component of polar milk fat globule membrane (MFGM) [52]. Milk fat globules (MFGs), the main form of lipids, are necessarily enveloped by the MFGM to be released into the alveolar lumen through exocytosis [53]. The main components of dairy fat are triglycerides (TGs) [54], which contain one glycerol molecule and three fatty acid molecules [55]. In the black module, the activation of the dicarboxylic acid transmembrane transporter activity term (GO:0005310) and dicarboxylic acid transport term (GO:0006835) provided dicarboxylic acids for the synthesis of TGs. In addition, the black module was positively correlated with milk protein. GO analysis evidenced that numerous terms in the black module were related to milk protein, including the regulation of the protein processing (GO: 0070613) and protein maturation (GO: 1903317; Figure 8A-1, File S1) terms.

## 5. Conclusions

We constructed, for the first time, a gene coexpression network related to 18 gene expression profiles in Holstein dairy cow mammary glands during six stages of lactation by transcriptome sequencing, characterized the genes’ temporal patterns, detected milk yield and the content of milk composition, and revealed the biological significance pathways and genes associated with milk yield and composition. The current study provides an important theoretical basis for the further molecular breeding of dairy cows.

## Figures and Tables

**Table 1:** Milk yield, milk lactose, milk fat and milk protein at different lactation stages (means ± SE).

Test Days	30 d	90 d	180 d	270 d	SEM	*p*
Milk yield (Kg)	32.65 ^b^	34.40 ^a^	31.12 ^c^	26.46 ^d^	0.13	<0.0001
Milk lactose (%)	5.05 ^b^	5.11 ^a^	5.04 ^b^	4.93 ^c^	0.01	0.0007
Milk fat (%)	3.40 ^d^	3.48 ^c^	3.58 ^b^	3.87 ^a^	0.02	<0.0001
Milk protein (%)	3.12 ^c^	3.15 ^c^	3.24 ^b^	3.32 ^a^	0.01	<0.0001

Note: ^a^, ^b^, ^c^ in the same row represent significant differences (*p* < 0.05).

**Table 2:** The number of genes in the eleven coexpression modules.

Module Colors	Red	Purple	Green	Black	Yellow	Pink	Blue	Turquoise	Brown	Magenta	Grey
**Node Number**	63	38	75	62	83	50	164	286	151	41	12,163

## Data Availability

The data presented in this study are available from Supplementary Materials.

## Supplementary Materials

The following are available online at https://www.mdpi.com/2076-2615/11/2/314/s1, Table S1: The primers used for qRT-PCR to validate the transcriptome sequencing, Table S2: Basic information of sequencing reads and bases, Table S3: Statistics of total reads mapping to the reference genome and quality parameters, Figure S1: Determination of soft threshold, File S1: The significant gene ontology (GO) terms in different modules, File S2: The significant Kyoto Encyclopedia of Genes and Genomes (KEGG) pathways in different modules.
