# Supplementary material for: Lactation Associated Genes Revealed in Holstein Dairy Cows by Weighted Gene Co-Expression Network Analysis (WGCNA)
Source: Animals (Basel). 2021 Jan 27;11(2):314. doi: 10.3390/ani11020314 (PMC7911360; doi:10.3390/ani11020314)
Supplement: Supplementary file 1 [file animals-11-00314-s001.zip › Supplementary tables and figures-animals-1082830.pdf]

animals

MDPI

Supplementary Materiasls

Lactation Associated Genes Revealed in Holstein Dairy Cows by Weighted Gene Co-expression Network Analysis (WGCNA)

Yongliang Fan, Abdelaziz Adam Idriss Arbab, Huimin Zhang, Yi Yang, Mudasir Nazar, Ziyin Han and Zhangping Yang

Table S1. The primers used for qRT-PCR to validate the transcriptome sequencing.

Gene	Forward Primers (5′–3′)	Reverse Primers (5′–3′)	Length (bp)	GenBank ID
β-actin	CATCCTGACCCTCAAGTA	CTCGTTGTAGAAGGTGTG	91	NM_173979.3
MAPK15	GAGGTCGTGGCCATCAAGAA	GTTCTCTGCCGGGATCACAT	144	NM_001046110.1
GALNT16	CTGCTCTGCAGGACCGA	ATTGGCGCGGATCTTCCTC	145	NM_001101127
IGFBP6	GACACTGAGATGGGTCCCTG	TCCGGTAGAAGCCCCTTTGG	124	NM_001040495.2
AGPAT2	CCATGAGCGTGATGACCGAT	CTGGATCGCCAGGTAGAAGG	137	NM_001080264.1
PVRIG	GGGTGGTTAAAGAGAGGCCA	CGTATGAGGATGAGCGGTCC	138	XM_024985330.1
SCD	TGCAGAAGTGGCTGGTATAAA	CTTCTACCCTCCATCAAATGTG	142	NM_173959
B4GALNT4	ACCGGGTTGGGGGATATAGT	GACCCTGAACGAGGGTTACA	122	XM_024987440.1
SOCS3	TCTGTCGGAAGACCGTCAAC	CTAAAGCGGGGCATCGTACT	104	NM_174466.2
SERPINE1	AAGAGCACCGTCCAGAGAGA	CCGCATCCTGAATTTCGCAG	124	NM_174137.2
SLC26A6	CATCGCTGTGGTTGGGTTTG	GATGCCCCCAAGGAGGTTAC	127	NM_001076852.2

Table S2. Basic information of sequencing reads and bases.

Sample	Raw Reads	Raw Bases	Clean Reads	Clean Bases	Valid Ratio (Base)	Q30	GC Content
A-−7d	57799386	7224923250	56999706	7123209028	98.59%	97.01%	50.00%
B-−7d	64119978	8014997250	62393630	7796965713	97.27%	95.84%	51.50%
C-−7d	58315516	7289439500	57579696	7195686299	98.71%	97.07%	50.50%
A-30d	60909716	7613714500	60088626	7509331405	98.62%	97.07%	49.00%
B-30d	56006056	7000757000	55369090	6919663405	98.84%	97.27%	47.50%
C-30d	56573916	7071739500	55890988	6984780894	98.77%	97.18%	48.00%
A-90d	61255240	7656905000	60490684	7559605202	98.72%	97.14%	48.50%
B-90d	61664866	7708108250	60994408	7622698912	98.89%	97.33%	47.50%
C-90d	59050772	7381346500	58314034	7287463274	98.72%	97.11%	49.00%
A-180d	73996266	9249533250	72947722	9117045311	98.56%	96.54%	48.00%
B-180d	67909316	8488664500	66910040	8362357880	98.51%	96.33%	49.00%
C-180d	75935580	9491947500	74705698	9336502305	98.36%	96.19%	50.00%
A-315d	64237588	8029698500	63029200	7877151037	98.10%	95.93%	51.00%
B-315d	66336970	8292121250	65276846	8158134238	98.38%	96.21%	50.00%
C-315d	71028854	8878606750	69794682	8722707128	98.24%	96.09%	50.00%

Table S3. Statistics of total reads mapping to the reference genome and quality parameters.

Sample	Total Reads	Total Mapped	Multiple Mapped	Uniquely Mapped
A-−7d	56999706	52922505 (92.85%)	2345801 (4.12%)	50576704 (88.73%)
B-−7d	62393630	58107310 (93.13%)	2745151 (4.40%)	55362159 (88.73%)
C-−7d	57579696	53221712 (92.43%)	2143133 (3.72%)	51078579 (88.71%)
A-30d	60088626	54908846 (91.38%)	4682452 (7.79%)	50226394 (83.59%)
B-30d	55369090	50950136 (92.02%)	5997124 (10.83%)	44953012 (81.19%)
C-30d	55890988	52255429 (93.50%)	4420084 (7.91%)	47835345 (85.59%)
A-90d	60490684	54988154 (90.90%)	5366330 (8.87%)	49621824 (82.03%)
B-90d	60994408	56242133 (92.21%)	7919400 (12.98%)	48322733 (79.22%)
C-90d	58314034	54192238 (92.93%)	2025697 (3.47%)	52166541 (89.46%)
A-180d	72947722	66121187 (90.64%)	8320673 (11.41%)	57800514 (79.24%)

B-180d	66910040	61243612 (91.53%)	4779597 (7.14%)	56464015 (84.39%)
C-180d	74705698	69431670 (92.94%)	3057989 (4.09%)	66373681 (88.85%)
A-270d	70550840	64298141 (91.14%)	6959543 (9.86%)	57338598 (81.27%)
B-270d	76857188	69765025 (90.77%)	4206727 (5.47%)	65558298 (85.30%)
C-270d	62867970	58484736 (93.03%)	2654781 (4.22%)	55829955 (88.81%)
A-315d	63029200	58052074 (92.10%)	2547563 (4.04%)	55504511 (88.06%)
B-315d	65276846	60355909 (92.46%)	2606294 (3.99%)	57749615 (88.47%)
C-315d	69794682	64782101 (92.82%)	2642781 (3.79%)	62139320 (89.03%)

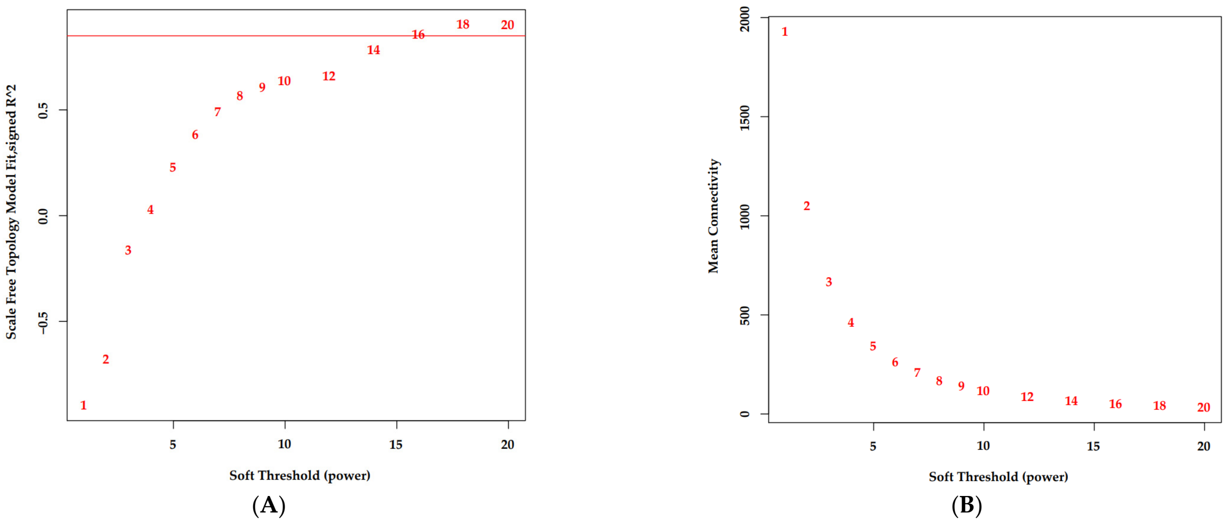

Figure S1. Determination of soft threshold. The abscissa represented the soft threshold. (**A**) Ordinate corresponded to the index of scale free network model. (**B**) The average link degree of each soft threshold.
